# Neurocutaneous Melanosis with Leptomeningeal Melanoma Involving Supratentorium and Infratentorium

**DOI:** 10.7759/cureus.3275

**Published:** 2018-09-10

**Authors:** Solly Thomas, Bimal Patel, Sunitha S Varghese, Selvamani Backianathan

**Affiliations:** 1 Radiation Oncology, Christian Medical College, Vellore, IND; 2 Pathology, Christian Medical College Hospital, Vellore, IND; 3 Radiation Oncology, Christian Medical College Hospital, Vellore, IND; 4 Radiotherapy, Christian Medical College, Vellore, IND

**Keywords:** neurocutaneous melanoma, congenital nevi, adjuvant hypofractionated radiotherapy

## Abstract

Neurocutaneous melanoma is a rare congenital syndrome associated with congenital melanocytic nevi with meningeal melanosis or melanoma. The disease is aggressive and has a high propensity for leptomeningeal metastases. We present the case history of a man with neurocutaneous melanoma managed with radical excision followed by hypofractionated adjuvant radiotherapy. One year, eight months later, he had a recurrence of the condition with leptomeningeal spread and was managed with re-excision of the recurrent lesion. Although our patient was disease-free for 20 months after the initial surgery, he survived only approximately five months after the second surgery, which reflects the associated poor prognosis of the disease.

## Introduction

Neurocutaneous melanoma (NCM) is a rare non-inherited embryonic neuroectodermal dysplasia [1]. Since its first description, only about 100 cases of neurocutaneous melanoma have been reported. Symptomatic NCM has a very poor prognosis, and chemotherapy and radiation therapy offer little benefit [2]. We report a case history and management of a teenaged boy with a large congenital nevus who presented with an intracranial melanoma, and we briefly discuss literature relevant to his case.

## Case presentation

An 18-year-old male presented to our institution in 2014 with headaches and vomiting for two weeks. A magnetic resonance image (MRI) of his brain showed a complex extra-axial dumbbell-shaped lesion with the epicenter in the left Meckel’s cave, extending anteriorly to the cavernous sinus and the cerebellopontine (CP) angle posteriorly with mass effect over the brainstem. He underwent a left retromastoid craniectomy and decompression of left CP angle lesion elsewhere. The histopathology was suggestive of malignant melanoma, and he was referred to our institution for further management.

His general physical examination revealed a deep gray-blue nevus over the left upper eyelid, extending to the frontal and temporal region. He had dysarthria, left upper motor neuron facial nerve palsy, and right hemiparesis with Grade 4 power in his right upper and lower limbs. The biopsy of the nevus over his left eyelid was reported as superficial, and the deep dermal dendritic melanocytosis with histological features was suggestive of a blue nevus.

**Figure 1:**
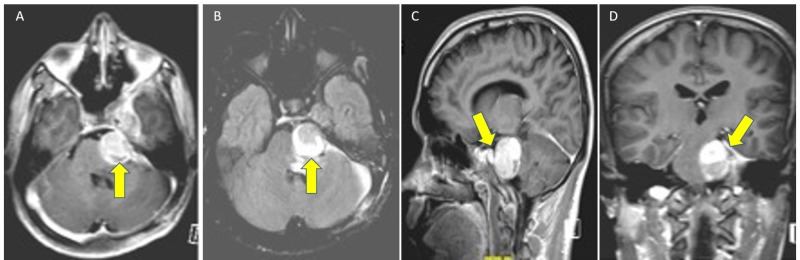
Magnetic resonance images (A) T1 Gado, (B) T2 Flair, (C) T1-weighted sagittal, and (D) T1-weighted coronal lobulated dumbbell-shaped extra-axial mass on the left of the posterior fossa along the left trigeminal nerve, widening of Meckel’s cave and displacing the cavernous sinus with indentation of regional pons.

The MRI of his brain showed a large contrast-enhanced extra-axial mass with solid and cystic components measuring 4 cm x 2 cm x 3 cm in the left cavernous sinus extending through the Meckel’s cave into the posterior fossa (Figure 1). A whole-body positron emission tomography-computed tomography (PET-CT) scan confirmed no extracranial disease. He underwent a left temporal craniotomy and zygomatic osteotomy, and we took an interdural middle cranial fossa approach for the radical excision of the tumor.

Perioperatively, we noted the blue nevus on the left side of the forehead in the ophthalmic distribution of the trigeminal nerve. The pigmentation extended into the subcutaneous tissue and galea. The diploe of the temporal bone was also pigmented. The entire temporal dural convexity was pigmented completely black as were the dural root sleeves of the trigeminal nerve and the lateral and medial walls of the cavernous sinus. The tumor was localized in the cavernous sinus and had a well-defined capsule surrounding the divisions of the fifth cranial nerve. It extended into the posterior fossa through Meckel’s cave. The tumor was completely removed via the cavernous sinus through an interdural approach.

**Figure 2:**
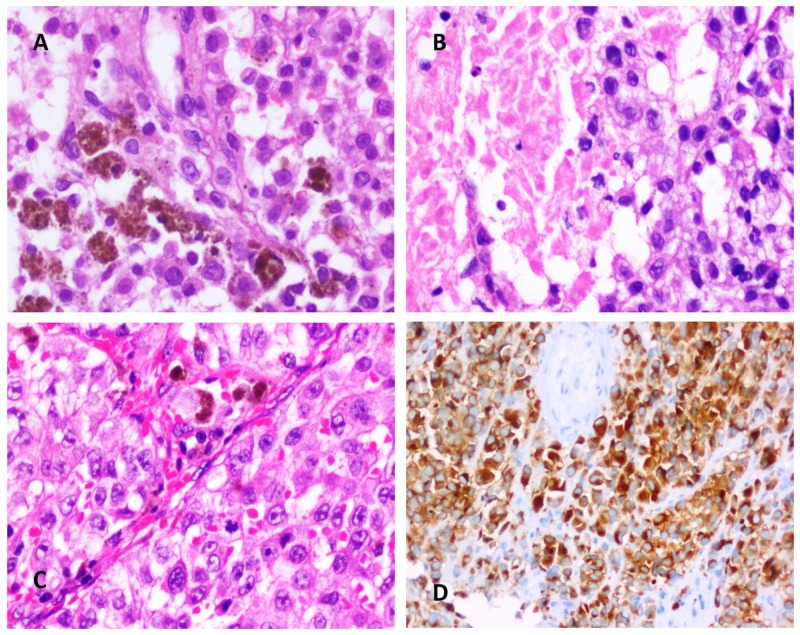
(A) Hematoxylin and Eosin (H&E) stained sections show sheets of medium-sized polygonal cells with moderately pleomorphic nuclei containing dispersed chromatin, macronucleoli, and moderate amounts of eosinophilic cytoplasm. Few cells contain intracytoplasmic melanin pigment (X400). (B) H&E-stained sections show tumor as described in A with an area of necrosis (X400). (C) H&E-stained sections show tumor as described in A (X200). (D) Immunoperoxidase staining for Melan A showing diffuse cytoplasmic staining in tumor cells (X200).

The surgical specimen revealed a tumor composed of sheets of moderately large polygonal cells with markedly pleomorphic nuclei with evidence of mitotic activity with foci of necrosis, and occasional cells with intracytoplasmic melanin (Figure 2). We also saw small segments of nerve containing ganglion cells with perineural deposits of melanin. The tumor cells showed diffuse positivity for S100 protein and Melan A. Occasional cells were positive for human melanin black (HMB)-45. The ki67/mib-1 was 20% to 25%.

Given the coexistent skin lesion reported as a blue nevus, neurocutaneous melanoma was considered as the provisional diagnosis. The postoperative hyperacute MRI showed no residual tumor. His symptoms gradually resolved after surgery. He received postoperative intensity modulated radiotherapy to the tumor bed (4950 cGy in 22 fractions to the planning target volume with a biologically effective dose [BED] of 60.39 Gy and an equivalent dose in 2 Gy fractions [EQD2] of 50.32). He was closely monitored during the follow-up period.

He presented again about 20 months later with recurrent symptoms of headaches for two months and diplopia and vomiting for two days. On examination, sensations over the V1, V2, and V3 dermatomes were reduced on the left side. There was masseter and temporalis muscle wasting on the left side with impaired blinking in both eyes. His spino-motor system and higher mental functions were found to be normal.

**Figure 3:**
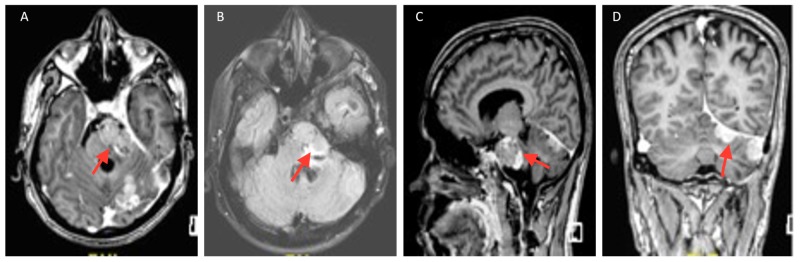
Magnetic resonance images (A) T1 Gado, (B) T2 Flair, (C) T1-weighted sagittal, and (D) T1-weighted coronal - multiple leptomeningeal and pachymeningeal T1 hyperintense areas were seen in both lobes of cerebral hemispheres.

A lobulated heterogenous signal intensity mass lesion was seen in the left CP angle and Meckel’s cave. The mass is predominantly isointense and weighted T1, and T2 images were hypointense (Figure 3). Various other similar intensity lesions were seen along the left tentorium suggestive of recurrent meningeal carcinomatosis.

The brain MRI with contrast showed a recurrent mass in the left Meckel’s cave extending into the CP angle compressing the brainstem. Another lesion of a similar nature was seen in the lateral aspect of the cerebellum. There was evidence of thickening and abnormal enhancement of the adjacent pachymeninges and leptomeninges suggesting disease recurrence with leptomeningeal spread. He was further evaluated with a CT of his thorax and abdomen which ruled out extracranial disease.

He underwent a left retromastoid suboccipital re-exploration and subtotal excision of the tumor. A postoperative CT scan of his brain showed no residual disease. However, the patient died five months after the second surgery.

## Discussion

NCM is a rare congenital syndrome, thought to represent an error in the morphogenesis of the embryonal ectoderm [1]. Most patients present with neurological symptoms in the first two years of life. NCM is defined by the presence of melanocyte deposits in the central nervous system along with large or multiple congenital nevi. The malignant transformation of the meningeal deposits occurs in approximately 2.3% of patients [3].

Our patient had a rare presentation of NCM with leptomeningeal melanoma manifesting in his second decade of life. A definitive criterion for the diagnosis of neurocutaneous melanoma was established by Kadonaga and Frieden in 1991 [1]. The patients with the highest risk of NCM have associated nevi in the head and neck region or over the dorsal spinal cord. Gadolinium-enhanced cranial MRI within the first four to six months of life is the modality of choice for the evaluation of NCM [2]. The neuroradiological appearance of melanocytic deposits consists of T1 hyperintensity and T2 hypointensity, most commonly seen over mesial temporal structures, the cerebellum or the leptomeninges. Our patient had a contrast-enhanced extra-axial mass in the region of Meckel’s cave which was hyperintense on T1 and hypointense on T2 weighted MRIs. The common neurological presentations of NCM are seizures or raised intracranial tension [2].

NCM has a bimodal distribution. The first peak usually occurs before age three, and the other peak occurs in the second and third decades of life. The risk factors for NCM are the presence of giant congenital melanocytic nevi, male sex, satellite nevi or multiple congenital melanocytic nevi, and nevi in the head and neck region. Our patient is an 18-year-old male presenting with a congenital nevus involving the left upper eyelid and frontal and temporal regions. The characteristic nevus that is associated with leptomeningeal melanosis is the nevus of Ota, which is a hyperpigmented lesion that involves the eyelid and adjacent skin and is associated with pigmentation of the scleral connective tissue. It most often involves the distribution of trigeminal nerve [2].

The usual histopathological features include marked cellular pleomorphism, the presence of mitoses, necrosis, and hemorrhage. On immunohistochemistry, the tumor cells show diffuse positivity for the HMB-45 antibody, S100 and Melan A, suggestive of melanocytic origin [4]. Melanomas do not stain for epithelial membrane antigen (EMA), and this differentiates it from meningioma. Our patient’s tumor also showed positivity for HMB-45 and S 100 and was negative for EMA [5]. It also had features suggestive of necrosis and increased mitotic activity with cellular atypia more favouring a malignant process.

Treatment options

Since NCM with leptomeningeal melanosis being a rare tumor, there are no specific guidelines for its management. Complete surgical removal of the intracranial tumor is the mainstay of management. The adjuvant treatment options include radiotherapy (focal, whole brain or craniospinal) depending on the extent of the disease at presentation [5]. Intrathecal and systemic chemotherapy was tried by various researchers with some benefit in metastatic melanoma, but not in primary leptomeningeal melanoma [6,7].

Adjuvant Radiotherapy

Adjuvant radiotherapy should be considered at a BED of >45 Gy, as the local control was suboptimal at doses <45 Gy EQD2 [8]. The survival reported in the literature ranges from a few days to three years [3], and our patient survived for 25 months after diagnosis.

Role of Targeted Therapy in Intracranial Melanoma

Forty percent to 60% of melanomas carry mutations in the BRAF protein pathway. Ninety percent of these mutations result in the substitution of glutamic acid for valine at codon 600 (BRAF V600E). Vemurafenib is a potent inhibitor of mutated BRAF. It has marked antitumor effects against melanoma with BRAF V600E mutation [9]. Dabrafenib is another BRAF inhibitor which has shown efficacy as a monotherapy in BRAF-mutated melanoma. Acquired resistance to BRAF inhibitor frequently develops through reactivation of the MAPK pathway. Hence, a combination of a BRAF inhibitor with an MEK inhibitor results in a significant delay in the emergence of the resistance. A combination of dabrafenib and trametinib showed a better progression-free survival compared to single-agent therapy with vemurafenib [10].

## Conclusions

We report a rare presentation of neurocutaneous melanosis with leptomeningeal spread presenting as a supra and infratentorial mass in the second decade of life. The patient was treated with radical resection and hypofractionated radiotherapy. There was local recurrence after 20 months which was treated with re-excision and had a survival of five months post treatment. Targeted therapy with BRAF inhibitors and MEK inhibitors may offer a promising treatment.

## References

[1] Kadonaga JN, Frieden IJ (1991). Neurocutaneous melanosis: definition and review of the literature. J Am Acad Dermatol.

[2] Acosta FL, Binder DK, Barkovich AJ, Frieden IJ, Gupta N (2005). Neurocutaneous melanosis presenting with hydrocephalus: case report and review of the literature. J Neurosurg.

[3] Hale EK, Stein J, Ben-Porat L (2005). Association of melanoma and neurocutaneous melanocytosis with large congenital melanocytic naevi—results from the NYU-LCMN registry. Br J Dermatol.

[4] Brat DJ, Giannini C, Scheithauer BW, Burger PC (1999). Primary melanocytic neoplasms of the central nervous systems. Am J Surg Pathol.

[5] Balakrishnan R, Porag R, Asif DS, Satter AM, Taufiq M, Gaddam SS (2015). Primary intracranial melanoma with early leptomeningeal spread: a case report and treatment options available. Case Rep Oncol Med.

[6] Le Rhun E, Taillibert S, Chamberlain MC (2013). Carcinomatous meningitis: leptomeningeal metastases in solid tumors. Surg Neurol Int.

[7] Paul MJ, Summers Y, Calvert AH, Rustin G, Brampton MH, Thatcher N, Middleton MR (2002). Effect of temozolomide on central nervous system relapse in patients with advanced melanoma. Melanoma Res.

[8] Rades D, Schild SE (2006). Dose response relationship for fractionated irradiation in the treatment of spinal meningeal melanocytomas: a review of the literature. J Neurooncol.

[9] Chapman PB, Hauschild A, Robert C (2011). Improved survival with vemurafenib in melanoma with BRAF V600E mutation. N Engl J Med.

[10] Robert C, Karaszewska B, Schachter J (2015). Improved overall survival in melanoma with combined dabrafenib and trametinib. N Engl J Med.

